# Flexible bed allocations for hospital wards

**DOI:** 10.1007/s10729-016-9364-4

**Published:** 2016-04-08

**Authors:** René Bekker, Ger Koole, Dennis Roubos

**Affiliations:** 1 Department of Mathematics, VU University Amsterdam, De Boelelaan 1081a, 1081 HV Amsterdam, The Netherlands; 2 HOTflo Company, Schoutlaan 26, 6002 EA Weert, The Netherlands

**Keywords:** Clinical capacity, Flexible bed allocation, Bed pooling, Earmarking, Queueing model, Optimization

## Abstract

Flexibility in the usage of clinical beds is considered to be a key element to efficiently organize critical capacity. However, full flexibility can have some major drawbacks as large systems are more difficult to manage, lack effective care delivery due to absence of focus and require multi-skilled medical teams. In this paper, we identify practical guidelines on how beds should be allocated to provide both flexibility and utilize specialization. Specifically, small scale systems can often benefit from full flexibility. Threshold type of control is then effective to prioritize patient types and to cope with patients having diverse lengths of stay. For large scale systems, we assert that a little flexibility is generally sufficient to take advantage of most of the economies of scale. Bed reservation (earmarking) or, equivalently, organizing a shared ward of overflow, then performs well. The theoretical models and guidelines are illustrated with numerical examples. Moreover, we address a key question stemming from practice: how to distribute a fixed number of hospital beds over the different units?

## Introduction

Inpatient beds are a critical capacity in the patient care process within a hospital. Traditionally, the clinical organization is according to medical disciplines, resulting in separate nursing units for, e.g., medicine, surgery, cardiology, obstetrics, neurology, gynaelogy. Over the years other classifications have been introduced, such as length of stay (e.g., short and long stay, see for example [29]), level of care (intensive, medium, special or normal care), or urgency (elective, urgent and emergent), each having organizational advantages. A disadvantage of a strict classification of inpatient beds is that this may result in small scale hospital units. Such small scale units suffer severely from the variability of health care processes [7]. More generally, it is well known that the efficiency of service systems often increases as the system becomes larger [32]. This is referred to as ‘economies of scale’ (abbreviated as EOS). Flexibility in bed usage is thus a key concept for an efficient management of beds, as has been recognized in, e.g., [4, 9, 12, 18], and is of fundamental importance for the increasing pressure to reduce costs.

**Table 1:** Pros and cons of different bed allocation policies

	Specialization	Flexibility	Prioritize	Accommodate	Management &
		& EOS	pat. types	diverse LOS	bed guarantees
Separate wards	++	–	+	+	++
Simple merging	–	++	–	–	–
Earmarking	+	+	+	+	+
Threshold	–	++	++	++	–

On the opposite, in manufacturing it has long been recognized that focus on a limited range of tasks improves efficiency. This principle of specialization advocates to divide capacity to patient groups with similar medical conditions, see, e.g., [13, 27, 30] and references therein for some health care related studies. The increasing focus on more complex cases further advocates to organize specialized hospital units, which is evidently necessary to some extent. A further disadvantage of flexibility is that this requires the medical staff, such as nurses, to be able to treat multiple patient types. This may require costly additional training efforts. Moreover, small wards guarantee personalized patient care and may improve work satisfaction and efficiency of nurses.

Apart from medical specializations and the potential improvements from economies of focus, there are some other issues with full flexibility. First, the overall performance may improve, but that may be at the expense of one type of patients. This may be unwanted in case that a particular patient type should be prioritized (e.g., receive specialized care). Related is the example in [9] of cardiac and thoracic surgery, where cardiac patients have priority over thoracic patients. Under their average delay constraints and taking the priority for cardiology into account, it follows that a combined unit would actually need more beds than two separate units. Second, the overall performance may even decrease in case of non-identical average service times (also referred to as average length of stay, abbreviated as ALOS). This observation goes back to [24]. In that case, patients with prolonged hospital stay block access for patients with high turnovers.

In this paper, we propose an intermediate organizational bed assignment that utilizes the efficiency gains of large systems and avoids the drawbacks mentioned above. More specifically, we consider the following bed allocation policies:

- *Separate wards*: Each patient type has dedicated beds.
- *Simple merging*: All patient types share all beds.
- *Earmarking*: Each patient type has dedicated (earmarked) beds, whereas all patient types share a joint ward of overflow with fully flexible beds.
- *Threshold policy*: All beds are fully flexible, but there is a hierarchy in admission of patient types. The most important (e.g., most urgent) patients are always admitted when beds are available, but other patient types are only admitted when the number of available beds exceeds some (prespecified) threshold.

The advantages and disadvantages of the different bed allocation policies are indicated in Table 1. These findings are further supported in the rest of the paper. Specialization refers to all benefits of having small scale units, such as specialized medical teams, single-skilled nurses and efficiency in task performance due to routine operations. Flexibility and EOS refer to all benefits of large systems, such as the ability to handle peaks in demand, flexibility in allocation of beds and flexibility in nurse rostering (see, e.g., Burke et al. [5]). Bed guarantees means that different patient types have allocated beds, making bed management significantly easier. Prioritization and the efficiency in accommodating patients with severely different LOS are further addressed in Section 4.2.

For large scale systems specialization often is a major requirement, leading to the distribution of beds over different medical units. The earmarking policy is then effective (see Section 4.3). At a smaller scale, i.e., within a single unit, further specialization might be unnecessary and the focus is rather on efficient bed usage and accommodation of different patient types (see Section 4.2).

### **Goals and contribution**

The issue of how to allocate partially flexible capacity for clinical wards has not yet been addressed in the literature. Therefore, our contribution is two-fold. First, we identify which structure of the bed allocation policy is appropriate for balancing between flexibility and the issues of large scale systems. For this structure we distinguish two cases that differ in system size, as they require a different approach.*Bed allocation for small scale systems*: at the unit level (like an ICU), the number of beds is shared by different patient groups. For instance, a patient group may represent a medical discipline, patients with a similar diagnosis, or similar level of urgency. As the sizes of the patient groups are small, specialization is inefficient whereas an earmarking policy often is less effective. In this setting, threshold policies are effective when there is a difference in priority for patient types, or patient types have an entirely different ALOS.*Bed distribution for large scale systems*: at the hospital level, the total number of staffed beds should be distributed over the different (often medical) units. To allow for flexible bed usage and avoid large-system size issues at the same time, earmarking is an effective policy. We see that some flexibility is sufficient to accommodate most of the peaks in bed demand. The beds at each ward are dedicated (earmarked) that can be handled by specialized medical teams, whereas the beds at the joint ward are flexible.

In the literature the commonly addressed question is ‘how many hospital beds?’ [4, 8]. In practice, the overall number of beds is limited due to the building construction and obtained licenses [12]. The typical question for hospital managers therefore is ‘how to distribute hospital beds?’. We provide rules of thumb based on square-root staffing for the distribution of the fixed number of total beds across units.The second contribution is that we provide models to support strategic and tactical decision making regarding ward sizes and the level of flexibility. Specifically, using these models, the exact number of beds and its allocation for the corresponding policy can be determined. For large scale systems, the performance of an earmarking policy can easily be calculated due to the product-form solution. To enhance application of threshold policies, the models are suitable for a form of decision support as well. We like to emphasize that well-founded hospital management of bed capacity requires quantitative models to visualize the impact of strategic management decisions and policies.

### **Queueing literature**

We now briefly review some of the basic queueing literature related to pooling. The term bed pooling is also often encountered in the literature when different units fully share their capacity. As mentioned, Smith and Whitt [24] seem to be the first to give counterexamples to show that full flexibility or resource sharing is not always beneficial. Another early paper supporting this from a qualitative perspective is Rothkopf and Rech [23]. In Mandelbaum and Reiman [20], the authors consider queueing networks in which both servers (beds) and queues can be pooled. They quantify the effect of pooling in terms of an efficiency index and show that pooling always helps in light traffic, but that pooling effects can go either way in heavy traffic. We refer to the references in [20] for the application of pooling in different application areas.

In the context of call centers, van Dijk and van der Sluis [25] gave some instructive examples where pooling is not beneficial and they proposed overflow pooling as an alternative. In overflow pooling the servers are dedicated to a queue, but they can serve customers from the other queue in case the server becomes idle. The concept of pooling is also related to skill-based routing in call centers. For instance, Wallace and Whitt [28] showed that “a little flexibility goes a long way”, meaning that only a few generalists are required to approach near optimal performance. In Chevalier et al. [6], the authors find that a 80/20 rule works well for a remarkably wide range of parameters. Here, the 80/20 rule means that 20 % of the staffing budget should be spent on flexible (multi-skilled) servers while 80 % should be spent on dedicated (single-skilled) servers. This already hints that flexibility and specialization can go hand in hand in hospital systems.

From a different angle, van Essen et al. [26] consider how departments should be clustered to benefit from scale effects. The authors take into account that not all departments can be clustered and that patients should not be spread over the hospital. Clustering is formulated as an optimization problem where blocking probabilities impose constraints. As the optimization problem is strongly NP-hard, the authors provide two heuristic approaches in addition to the exact formulation.

### **Organization**

The paper is organized as follows. We introduce the general model and assumptions in Section 2. The bed allocation policies and its performance analysis are discussed in Section 3. In Section 4 we show numerical results. The allocation of beds over different patient groups within a unit is studied in Section 4.2. In Section 4.3 we consider the distribution of beds over different units at the hospital level. Section 5 concludes.

## Model

We analyze the patient flow through the clinical wards in the spirit of the Erlang loss model. The aim of this model is to support managerial decision making at the strategic and tactical level. We first introduce the main assumptions in Section 2.1 and then formally define the model in Section 2.2.

### Basic assumptions

The assumptions of the model are based on the data analysis in [4] of 24 hospital wards of the VU medical center in addition to our experience with other Dutch hospitals.

#### **Arrival process**

The model assumes that patients arrive according to a Poisson process. This has been widely accepted for urgent patients, see for example [34]. Surprisingly, the number of elective admissions varies significantly as well. This variation can even be larger than the variation in urgent admissions [4, 21]. The Poissonian assumption therefore seems a reasonable approximation for the elective admission process (see also [31]).

#### **Length of stay**

The model assumes that the lengths of stay (abbreviated as LOS) are independent and identically distributed for each patient type. This seems an appropriate assumption as long as the patient mix and medical practice do not change. In practice, deviations from this assumption can occur, as the LOS may be affected by the level of congestion and delays in the care chain. In some cases we further assume, for mathematical convenience, exponentially distributed LOS. This often slightly underestimates the amount of variability present, but the impact on the results is typically very small (see Section 4.1).

#### **Beds**

The capacity of a unit is based on the number of operational beds. The number of operational beds is important for the distribution of budgets and is generally constant and evaluated on a yearly basis. The actual number of staffed beds may fluctuate slightly, but this rather is at an operational or tactical level.

#### **Bed blocking**

The model assumes that patients are blocked and lost from the system in case all appropriate beds are occupied. For urgent patients this means ambulance diversions and reallocation of patients at the Accident & Emergency department (A&E). For elective patients, unavailability of beds implies canceled admissions or surgeries. Such patients are often rescheduled, but this may affect the admissions of patients from the waiting list. As a rough approximation, we consider the rescheduled patients as new admissions.In Dutch hospitals, the waiting time at A&E departments for inpatient beds is usually short, whereas the fraction of transfers to other hospitals due to unavailability of beds is significant (estimated at about 10 %). In addition to our experience with Dutch hospitals, where excessive waiting for beds is uncommon, we chose to incorporate blocking. In the literature, delay models for bed capacity have also been proposed [7, 8]. Note that for the classical models, there is a direct relation between the probability of waiting (delay model) and the blocking probability (loss model). The delay models typically do not take flexible bed allocations into account. We refer to [19] where routing policies from emergency departments to internal wards are addressed in an asymptotic queueing framework.

### Model and notation

We consider the allocation of beds for *J* types of patients. A patient type typically refers to a medical discipline or to a specific diagnosis group. Patients of type *j* are assumed to arrive according to a Poisson process with rate *λ*_*j*_, *j* = 1,…, *J*. Denote the overall arrival rate by $\lambda = {\sum }_{j} \lambda _{j}$. Let the LOS of type *j* be denoted by *S*_*j*_ with mean ${\mathbb {E}} S_{j}$, *j* = 1,…, *J*. The traffic intensities are then $\rho _{j} :=\lambda _{j} {\mathbb {E}} S_{j}$. In case the LOS of type *j* is exponentially distributed, we let *μ*_*j*_ denote the corresponding rate.

The total number of beds available is *N*. There is no waiting room for patients. This means that when a patient arrives and all beds are occupied, the arriving patient is refused, see Section 2.1. However, patients can also be refused in other situations. For instance, when each ward has its own number of beds (say *N*_*j*_, with ${\sum }_{j} N_{j} = N$), patients are also refused when the preferred ward is fully occupied.

A major performance measure for clinical wards is the long-run fraction of refused admissions, also called loss or blocking probability. Let *b*_*j*_ denote the fraction of refused admissions (blocked patients) of type *j*. The weighted total fraction of refused admissions is given by $b_{\text {tot}} = {\sum }_{j=1}^{J} (\lambda _{j}/ \lambda ) b_{j}$. Let *α*_*j*_, *i* = 1,…, *J*, be the relative value for patients of type *j*. This reflects different levels of priorities for the patient groups that might be caused by, e.g., urgency or strategic focus of the hospital. Let **c** = (*c*_1_, . . . , *c*_*J*_) be fixed, where *c*_*j*_ is the weight of the loss fraction *b*_*j*_ in the objective function. For instance, in case *c*_*j*_ = *α*_*j*_ *λ*_*j*_/*λ* the objective is to minimize the weighted loss fraction that takes the relative values *α*_*j*_ into account (*b*_tot_ is then minimized in case *α*_*j*_≡1). Our objective is to minimize a linear combination of the *b*_*j*_’s, i.e.,

1 $$ \min b({\mathbf{c}}), \qquad \text{with} \quad b({\mathbf{c}}) = \sum\limits_{j=1}^{J} c_{j} b_{j}. $$

Another example is the case in which the loss fraction for type *j* should be bounded by $b^{\max }_{j}$, *j* = 1,…, *J*. For example, $b^{\max }_{j}$ may represent the loss fraction before a reallocation of beds. The optimization problem then reads

$$\begin{array}{@{}rcl@{}} & \min b({\mathbf{c}}) & \\ & \hspace{+2em} \text{s.t.} b_{j} \le b^{\max}_{j}, & \qquad j=1,\ldots,J. \end{array} $$

The Lagrange relaxation of this problem is

$$\min b({\mathbf{c}}) + \sum\limits_{j=1}^{J} \gamma_{j} (b_{j} - b^{\max}_{j}), $$

which is again a linear combination of *b*_*j*_’s; take *c*_*j*_ + *γ*_*j*_, *j* = 1,…, *J*, as coefficients in Eq. 1.Our main performance measure is the loss fraction, reflecting the quality of the care process. Due to PASTA^1^, the loss fraction is equivalent to the fraction of time during which no bed is available for a certain patient type (bed blocking).

Another important performance measure focusing on efficiency is the occupancy rate. In case of only dedicated beds, by Little’s law, the occupancy (in %) for type *j* is given by

2 $$ \frac{\rho_{j} (1-b_{j})}{N_{j}} \times 100 \%. $$

Since the number of shared beds can differ for different patient types, it is not always clear how the occupancy should be determined (i.e., what the appropriate value for the denominator of Eq. 2 is). However, as the arrival process is assumed to be exogenous, a decrease in the loss fraction directly implies an increase in the average number of occupied beds of the particular type (numerator of Eq. 2). For conciseness and ease of presentation, we only give the loss fraction throughout the paper.

## Bed allocations and analysis

In this section, we describe the bed allocation policies (Section 3.1) and consider their performance analysis (Section 3.2). Denote the number of type *j* patients present at an arbitrary arrival epoch by *x*_*j*_, *j* = 1,…, *J*, with *x* = (*x*_1_,…, *x*_*J*_) the corresponding vector.

### Bed allocations

The bed allocation strategies differ by the rule used for accepting newly arriving patients.

#### **Separate wards**

This policy corresponds to the situation in which each patient type has dedicated beds, i.e. has its own ward. Let *N*_*j*_ be the number of beds at ward *j*, with ${\sum }_{j=1}^{J} N_{j} = N$. An arriving patient of type *j* is admitted if and only if *x*_*j*_<*N*_*j*_.

#### **Simple merging**

This corresponds to fully join the different wards. An arriving patient (of either type) is now admitted in case ${\sum }_{j=1}^{J} x_{j} < N$ and refused otherwise.

#### **Earmarking beds**

This policy is useful to guarantee a certain number of beds for each type of patients in addition to a shared ward of overflow. We assume that *M*_*j*_ beds are reserved for patients of type *j*, with ${\sum }_{j} M_{j} \le N$. In case all beds for type *j* are occupied there is a ward of overflow that is shared by all patient types. The size of this joint ward is ${M_{\text {joint}}} = N - {\sum }_{j=1}^{J} M_{j}$. In the remainder, the earmarking policy with bed allocation *M*_1_,…, *M*_*J*_ is denoted by (*M*_1_,…, *M*_*J*_). An arriving patient of type *j* is now admitted in case there is a bed available among the allocated (earmarked) beds of type *j* or at the joint ward, and refused otherwise. For this policy, the earmarked beds should always be used as much as possible. This means that if the ward of overflow is full, it should be checked if it is possible to transfer a patient from the ward of overflow to a dedicated bed (in particular when a new patient arrives). From the above considerations, we can now state that an arriving patient of type *j* is admitted if and only if

$$x_{j} < M_{j} + {M_{\text{joint}}} - \sum\limits_{i \neq j} (x_{i} - M_{i})^{+}, $$

where (*x*)^+^ = max(*x*,0). Here, (*x*_*i*_−*M*_*i*_)^+^ represents the number of beds of the joint ward occupied by patients of type *i*.

The earmarking policy may be considered as an intermediate option between separate wards and simple merging. In case ${\sum }_{j} M_{j} = N$ the policy of earmarking reduces to the situation of *J* separate wards, whereas in case *M*_*j*_≡0 this bed allocation policy corresponds to simple merging.

#### **Threshold policies**

There can be a hierarchy in the admission of patients. To reserve a number of beds for patients with high priority we employ a threshold policy. For type *j* there is a threshold value *T*_*j*_ that represents a maximum on the number of occupied beds for which patients of type *j* are admitted. More specifically, an arriving patient of type *j* is admitted in case ${\sum }_{i} x_{i} < T_{j}$. Note that the patients of highest priority have *T*_*j*_ = *N*. The threshold policy with thresholds *T*_1_,…, *T*_*J*_ is denoted by (*T*_1_,…, *T*_*J*_).

#### **Optimal policy**

The main aim of the optimal policy is to compare the performance of the other proposed policies to best achievable values in case of fully dynamic admission control. Hence, it provides a benchmark for what is ideally possible and allows to evaluate the relative performance of the corresponding policy. Specifically, the optimal admission policy minimizes the objective function *b*(**c**). This implies that upon arrival of each type of patient, given the number of patients of each type present *x*, it is decided whether the patient is admitted or refused. Such a policy might be difficult to implement in a hospital, unless bed occupancy is digitally registered in real time.

### Performance analysis

Roughly speaking, the performance models can be classified in three categories, as addressed below. Some structural properties are discussed in Section 3.3.

#### **Separate wards and simple merging**

For the cases of separate wards or simple merging, the performance can be immediately obtained using the Erlang loss model. The blocking probability or loss fraction for separate ward *i* reads

$$b_{i} = B(\rho_{i}, N_{i}) = \frac{\rho_{i}^{N_{i}}/N_{i}!}{{\sum}_{k=0}^{N_{i}} {\rho_{i}^{k}}/k!}. $$

The total traffic load for the *J* type of patients equals $\rho = {\sum }_{j} \rho _{j}$. Using the Erlang loss formula again yields *b*_*i*_ = *B*(*ρ*, *N*) for all *i*∈{1,…, *J*} in case of simple merging.

#### **Earmarking beds**

A closed-form result for the number of patients present and the loss fraction can also be derived for the policy of earmarking beds. Let *M*_*i*_, *i* = 1,…, *J*, and *N* be fixed, and assume for the moment that the LOS follows an exponential distribution, and let *x*(*t*) denote the vector of the number of patients at time *t*. The stochastic process {*x*(*t*), *t*≥0} then clearly is a Markov process with state space $\mathcal {S} = \{ x \in \mathbb {Z}_{+}^{J} : x_{j} \le M_{j} + {M_{\text {joint}}} - {\sum }_{i \neq j} (x_{i} - M_{i})^{+}, j = 1, \ldots , J \}$, see Remark 3.1 for an alternative representation. The transition rates *q*(*x*, *x* ^′^) are given by

$$q(x,x^{\prime}) = \left\{ \begin{array}{ll} \lambda_{i}, & x^{\prime} = x + e_{i}, \quad x + e_{i} \in \mathcal{S}, \\ x_{i} \mu_{i} , & x^{\prime} = x - e_{i}. \end{array} \right. $$

Let *π*(*x*) denote the stationary distribution of *x*(*t*), which has the following product form:

$$\pi(x) = G^{-1} \prod\limits_{j=1}^{J} \frac{\rho_{j}^{x_{j}}}{x_{j} !}, $$

where $G = {\sum }_{x \in \mathcal {S}} {\prod }_{j=1}^{J} \rho _{j}^{x_{j}}/(x_{j} !)$ is the normalizing constant. This result can immediately be derived by verifying that *π*(*x*) satisfies the detailed balance equations

$$\pi(x) \lambda_{i} = \pi(x+e_{i}) (x_{i} + 1) \mu_{i}, \qquad x, x+e_{i} \in \mathcal{S}. $$

To obtain the fraction of refused admissions, define the sets $\mathcal {S}_{j} = \{ x \in \mathcal {S} : x_{j} = M_{j} + {M_{\text {joint}}} - {\sum }_{i \neq j} (x_{i} - M_{i})^{+} \}$ for *j* = 1,…, *J*. Using PASTA,we have $b_{j} = {\sum }_{x \in \mathcal {S}_{j}} \pi (x)$.

Finally, we note that the product-form result is insensitive to the LOS distribution, see Bonald [2] and references therein. Hence, we only require the average length of stay to determine the performance of the earmarking policy without assuming exponential LOS.

#### *Remark 3.1*

Note that the policy of earmarking can also be interpreted as a special case of a loss network, see, e.g., Kelly [15]. Let $\mathcal {P}(A)$ be the power set of *A*. The loss network then consists of *J* routes and 2^*J*^ links (the number of elements of $\mathcal {P}(\{1,\ldots ,J\})$). A call (patient) on route *r* then uses all links for which $r \in \mathcal {P}(\{1,\ldots ,J\})$. The number of circuits on link $I \subseteq \mathcal {P}(\{1,\ldots ,J\})$ equals ${\sum }_{j \in I} M_{j} + {M_{\text {joint}}}$. The state space, also giving the capacity constraints, can thus be written as $\mathcal {S} = \{ x \in \mathbb {Z}_{+}^{J} : {\sum }_{j \in I} x_{j} \le {\sum }_{j \in I} M_{j} + {M_{\text {joint}}}, \forall I \subseteq \mathcal {P}(\{1,\ldots ,J\})\}$.

#### **Optimal and threshold policies**

Contrary to the allocation policies of separate wards, simple merging and earmarking, there are no closed-form results for the performance of the optimal and threshold policies. For the optimal policy, we use dynamic programming to find the policy that minimizes the relative costs *b*(**c**). A similar iterative procedure, based on dynamic programming, can be used to determine the performance of threshold policies. For these policies, we require that the LOS follows an exponential distribution.

First, consider the optimal policy. The state space clearly is $\mathcal {S} = \{ x \in \mathbb {Z}_{+}^{J} : {\sum }_{j} x_{j} \le N \}$. We use uniformization and first rescale time such that ${\sum }_{j} \lambda _{j} + \max _{j} \{ \mu _{j} \} N = 1$. We associate costs when an arriving patient of type *j* is refused, representing the relative values in loss fractions. More specifically, we associate costs *α*_*j*_/*λ*; costs then represent loss fractions in case *α*_*j*_ equals 1. We note that the factor 1/*λ* in the costs is due to the fact that the average costs in the dynamic programming formulation represent average costs per time unit, whereas we are interested in customer averages instead of time averages.

The dynamic programming value function *V*_*n*_ at the *n*th epoch can then be determined by

$$\begin{array}{@{}rcl@{}} V_{n+1}(x) &=& \sum\limits_{j=1}^{J} \lambda_{j} \min \{ V_{n}(x+e_{j}), V_{n}(x) + \alpha_{j}/\lambda \} \\ & & + \sum\limits_{j=1}^{J} \mu_{j} x_{j} V_{n}((x-e_{j})^{+})\\&& + \left( 1-\sum\limits_{j=1}^{J} \left( \lambda_{j} + \mu_{j} x_{j} \right) \right) V_{n}(x), \end{array} $$

where we use the convention that *V*_*n*_(*x*) = *∞* for $x \notin \mathcal {S}$. Here, the first term represents an arrival, the second a departure, and the third term is due to uniformization. We note that at an arrival there is a decision to make. Either the patient of type *j* is accepted and the system moves to state *x* + *e*_*j*_, or the patient is refused and the systems stays in state *x* and incurs costs *α*_*j*_/*λ*. The minimal long-run average costs and the optimal policy can be found using value iteration.

We now turn to the performance analysis of a given policy, e.g., the threshold policy. For convenience, we also apply value iteration to determine the long-run average costs. Let *π* be a deterministic policy and define *π*(*j*, *x*) to be one in case an arriving patient of type *j* finding *x* patients present is admitted and let *π*(*j*, *x*) be zero otherwise. The value function $V^{\pi }_{n}$ at the *n*th epoch for policy *π* can then be determined by

$$\begin{array}{@{}rcl@{}} V^{\pi}_{n+1}(x) &=& \sum\limits_{j=1}^{J} \lambda_{j} \left( \pi(j, x) V_{n}(x+e_{j})\right.\\&& \left.+ (1-\pi(j, x))(V_{n}(x) + \alpha_{j}/\lambda) \right) \\ & & + \sum\limits_{j=1}^{J} \mu_{j} x_{j} V_{n}((x-e_{j})^{+})\\&& + \left( 1-\sum\limits_{j=1}^{J} \left( \lambda_{j} + \mu_{j} x_{j} \right) \right) V_{n}(x). \end{array} $$

We determine the value function and long-run average costs using value iteration again.

### Structural properties

In this part, we discuss a number of structural properties of the bed allocation policies.

(i) The optimal and threshold policies coincide in case the ALOS of the different patient types are identical (and the LOS is exponentially distributed), as can also be observed from the first example in Section 4.1. This is easy to explain by noting that the bed occupancy can then be modeled as a one-dimensional birth-and-death process. Since the ALOS are identical, the decision to accept or refuse an arriving patient now only depends on the available number of beds, and is independent of the type of patients present. This results can already be found in Lippman [17].
(ii) In the setting of call centers, Gurvich et al. [10] and Koçağa and Ward [16] have considered (partly) comparable control problems for Erlang C and Erlang A models, respectively. The authors show that threshold policies are asymptotically optimal, i.e. the limiting control scheme is of a threshold type for a sequence of systems with increasing arrival rates. Although the models are slightly different and the analysis involves an asymptotic framework, this supports the idea that threshold policies should work well in many practical situations.
(iii) In some cases, the patient groups can be indexed according to a priority list based on the values of *α*_*j*_ *μ*_*j*_. In case of two patient classes and *μ*_1_≥*μ*_2_ and *α*_1_≥*α*_2_ (and thus *α*_1_ *μ*_1_≥*α*_2_ *μ*_2_) it holds that if it is optimal to accept patient type 2 in some state, then it is also optimal to accept patient type 1, see Altman et al. [1]. A formal proof of a stronger result seems rather involved (see also [1]), and the structure of the optimal policy may differ, see [22, Example 3].
(iv) The priority list discussed above can be directly used to determine parameter values for threshold and earmarking policies. Again, without loss of generality, let *α*_1_ *μ*_1_≥⋯≥*α*_*J*_ *μ*_*J*_. For threshold policies, it can then be argued that *N* = *T*_1_≥⋯≥*T*_*J*_, also see [1]. For earmarking, we can directly conclude that *M*_*J*_ = 0, as class *J* needs no protection from other classes.

## Results on bed allocations

For determining suitable bed allocation policies, we need to consider two different cases that are related by the size of the system under consideration. Small scale systems tend not to suffer that severely from multi-skilled staffing issues, and are treated in Section 4.2. Multi-skilled staffing is only partly possible in large scale systems, thereby limiting the type of control. Large scale systems and the distribution of beds over the different units is discussed in Section 4.3. To clarify drawbacks related to full flexibility (holding for both small and large scale systems), we start with two instructive examples in Section 4.1.

### Instructive examples

We consider two cases in which differentiating between patient types might be desirable.

#### **Example I: Specialized care**

Consider two types of patients in which one type is of specific interest, e.g., it receives specialized care. Assume that the ALOS of both patient types is 4 days, i.e., *μ*_1_ = *μ*_2_ = 0.25, which roughly equals the ALOS at an Intensive Care (see also [4]). Let *λ*_1_ = 5 and *λ*_2_ = 2, yielding *ρ*_1_ = 20 and *ρ*_2_ = 8.

Now, assume that *N*_1_ = 20 and *N*_2_ = 12, such that *N* = 32. In that case, the loss fraction for type 1 and 2 patients are 15.9 % and 5.1 %, respectively, with a weighted average loss fraction of *b*_tot_ = 12.8 *%*. The difference in loss fractions may be a deliberate choice due to, for instance, the specialized care of type 2. Motivated by economies of scale, the bed allocation policy may be changed into simple merging. In that case, the loss fraction for both type of patients becomes 6.6 %. Hence, the average performance improves, but type 2 (specialized care) is negatively affected.

It is possible to prioritize type 2 patients using one of the three alternative bed allocation policies. The relative importance of type 2 is then quantified by *c*_*j*_ (or *α*_*j*_), *j* = 1,2. In general, it is not directly clear how to value this relative importance, unless the weights are identical. Using different weight combinations, the hospital manager obtains valuable insights to make this trade-off.

The case in which both patient types are equally important (*α*_1_ = *α*_2_) is trivial, since the ALOS of both types are also identical and the optimal policy is then simple merging. Consider now the situation that the hospital manager decides that the loss fraction of type 2 should be well below 5.1 % such that type 2 also benefits from the reallocation of beds. In case the value of type 2 patients is twice the value of type 1, i.e. *α*_2_ = 2*α*_1_, the optimal values for the three policies can be found in the first part of Table 2, where the parameters of the earmarking and threshold policy are chosen such that *b*(**c**) is minimized under the corresponding policy. An earmarking policy (*M*_1_, *M*_2_) denotes that *M*_*i*_ beds are dedicated to class *i*, *i* = 1,2, whereas the remaining beds, *N*−*M*_1_−*M*_2_ are fully flexible. Note that *b*_2_ is still above 5.1 % for the optimal earmarking policy. To further decrease the value of *b*_2_ at least 8 or 9 beds should be earmarked for type 2, see the second part of Table 2. The optimal and threshold policies thus outperform earmarking.

**Table 2:** Loss fractions in % for case I; first part corresponds to optimal values in case *α*_2_ = 2*α*_1_, the second part to some earmarking policies

	*b*_1_	*b*_2_	*b*_tot_	*b*(**c**)
Separate wards (20, 12)	15.89	5.14	12.82	14.29
Simple merging	6.65	6.65	6.65	8.55
Earmarking (0, 3)	6.66	6.64	6.65	8.55
Threshold (31, 32)	9.97	1.99	7.69	8.26
Optimal	9.97	1.99	7.69	8.26
Earmarking (0, 8)	8.42	5.12	7.48	8.94
Earmarking (16, 8)	8.40	5.20	7.49	8.97
Earmarking (0, 9)	9.70	4.29	8.15	9.38
Earmarking (16, 9)	9.68	4.37	8.16	9.41

Consider the case that (0,9) is a preferable earmarking allocation (e.g., it is optimal for *α*_2_ = 4*α*_1_). In that case, only 9 beds require single-skilled staff and 23 beds require multi-skilled staff. The limited number of multi-skilled staff (one of the main advantages) can then be exploited by choosing a much larger value for *M*_1_ with only a minor loss in performance. For instance, in the second part of Table 2 can be found that the difference in performance of (0, 8) and (16, 8) and (0, 9) and (16, 9) is negligible.

**Fig. 1:**
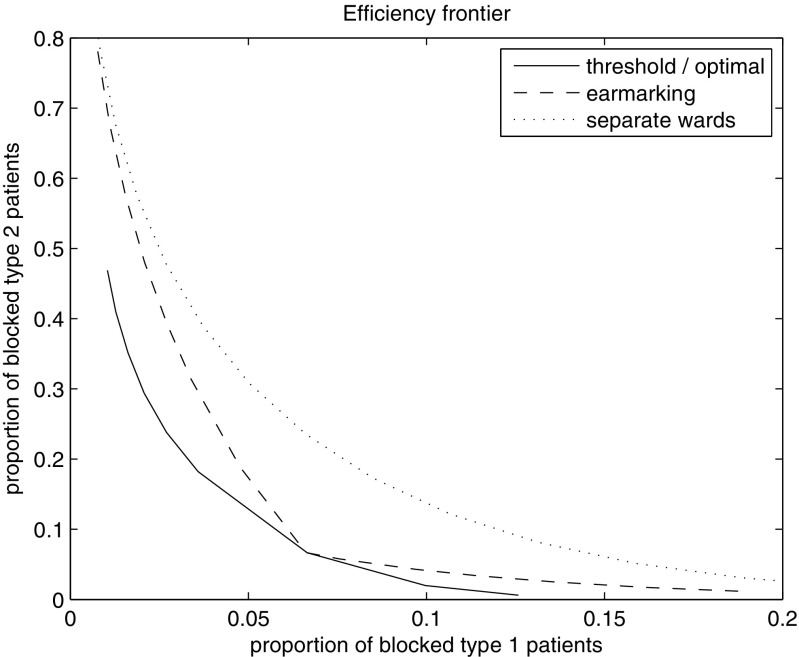
Efficiency frontier in case of specialized care at one ward

Alternatively, the possible optimal combinations of loss fractions for the three policies can be depicted by the efficiency frontier, see Fig. 1. The values on this line give combinations of *b*_1_ and *b*_2_ that are optimal for the considered policy class. From Fig. 1, it follows that the threshold and optimal policy coincide (see also Section 3.3) and that they (slightly) outperform earmarking especially for highly unbalanced loss fractions. In turn, earmarking outperforms separate wards, in particular for non-extreme blocking probabilities. Note that given the practical disadvantages of the threshold and optimal policy, it may be preferable to apply earmarking in some practical scenario’s.

#### **Example II: Patient types with different ALOS**

Consider two types of patients with a large difference in ALOS. As an illustration, assume that the ALOS of type 2 patients is 10 times as large as the ALOS of type 1 patients; we take *μ*_1_ = 1 and *μ*_2_ = 0.1. Let *λ*_1_ = 20 and *λ*_2_ = 2 such that the traffic loads are identical, i.e., *ρ*_1_ = *ρ*_2_ = 20.

The current bed allocation is often determined based on historically acquired privileges. For instance, assume that *N*_1_ = 27 and *N*_2_ = 17, such that *N* = 44. In that case, the loss fraction for type 1 and 2 patients are 2.7 % and 25.6 %, respectively. This yields an average loss fraction of *b*_tot_ = 4.8 *%*. Motivated by economies of scale, the bed allocation policy may be changed into simple merging. However, using the Erlang loss model, the loss fraction then turns out to increase to 6.5 %. Similar results in a different setting can be found in [25], indicating that simple merging does not necessarily work well in case of patient groups with a large difference in ALOS.

#### *Remark 4.1*

Since the load is identical for both types of patients it could be suggested to equally divide the number of beds over the two wards, that is *N*_1_ = *N*_2_ = 22. In that case, the loss fraction is 10.7 % for both patient types, which is much higher than the average of 4.8 % in case of allocation policy (27, 17). We note that the optimal bed allocation for separate wards in terms of minimal weighted average loss fraction is (30, 14) yielding an average loss fraction of 4.1 %.

For the moment, let us assume that both patient types are of equal importance, i.e., *α*_1_ = *α*_2_. The loss fractions (in %) for the different bed allocation policies can be found in Table 3. Note that the loss fraction of type 2 is well above 25 % for all optimal policies (except simple merging). This evidently follows from the large ALOS of type 2 and the fact that type 1 and 2 are of equal relative importance.

**Table 3:** Loss fractions in % for various policies in case II

	*b*_1_	*b*_2_	*b*_tot_
Separate wards (27, 17)	2.68	25.57	4.76
Simple merging	6.46	6.46	6.46
Earmarking (28, 0)	1.10	29.30	3.66
Threshold (44, 38)	1.22	26.67	3.53
Optimal	0.97	27.97	3.43

**Fig. 2:**
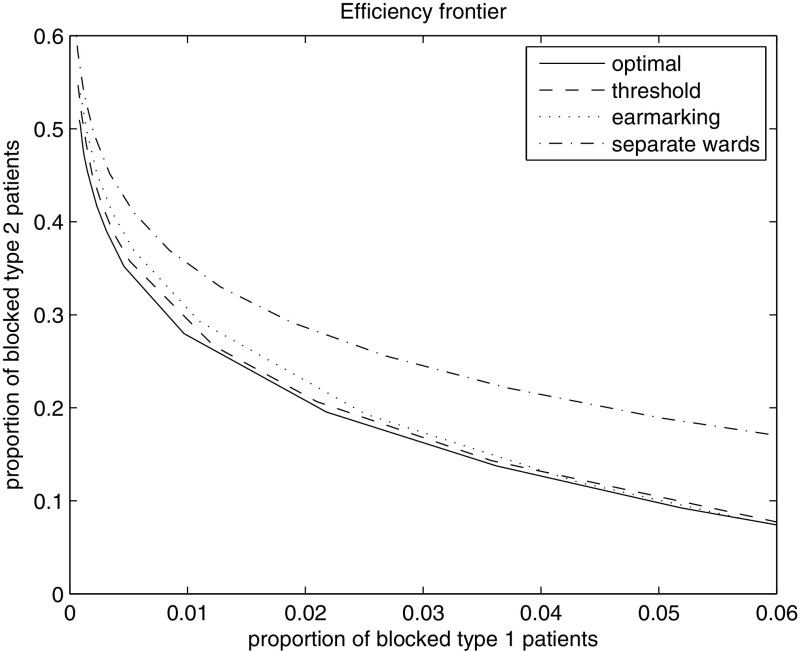
Efficiency frontier for two wards with different ALOS

The impact of changing the relative importance (i.e. *c*_*j*_, *j* = 1,2) can be seen using the efficiency frontier, see Fig. 2. In this example, both threshold policies and earmarking perform nearly as well as the optimal policy. It can also be observed that both type 1 and 2 may benefit from a different bed allocation compared to separate wards if the blocking probability of type 2 is not too large.

#### **LOS distribution**

For the analysis of the threshold and optimal policy, we assumed that the LOS is exponentially distributed. In practice we sometimes observe that the lognormal distribution gives a better fit for the length of stay. To investigate the sensitivity of our approach to the lognormal LOS distribution we have run several simulation experiments. The average blocking probability for lognormally distributed LOS is obtained using 100M events divided among 25 sub runs so that a confidence interval for the average blocking probability can be obtained using the student’s t distribution. The confidence was found to be such that the obtained blocking probabilities are accurate up to two decimal places.

The parameters of the lognormal distribution, denoted by *μ* and *σ* ^2^, are chosen such that the ALOS remains the same, whereas we varied the coefficient of variation. Specifically, the expectation and the variance of a lognormal random variable *X* are

$${\mathbb{E}}(X) = e^{\mu + \frac{1}{2} \sigma^{2}}, \qquad Var(X) = (e^{\sigma^{2}} - 1) e^{2\mu + \sigma^{2}}. $$

Hence, the squared coefficient of variation is ${c^{2}_{X}} = e^{\sigma ^{2}} - 1$. Now, we vary *σ* ^2^ (or ${c^{2}_{X}}$) and take *μ* = ln(ALOS)−*σ* ^2^/2. As mentioned, there is no difference in results between exponentially and lognormally distributed LOS for the policies of separate wards, simple merging and earmarking. The impact only becomes visible for the threshold and optimal policy. We have run experiments for the settings as in Examples I and II. The results are shown in Table 4.

**Table 4:** Loss fractions of types 1 and 2 in % for the threshold and optimal policy for lognormal LOS

	Example I		Example II	
LOS distr.	threshold (31, 32)	optimal	threshold (44, 38)	optimal
Exp.	(9.97, 1.99)	(9.97, 1.99)	(1.22, 26.67)	(0.97, 27.97)
LogN(*μ*, 0.05)	(9.96, 1.99)	(9.97, 1.99)	(1.38, 26.85)	(1.09, 28.14)
LogN(*μ*, 0.1)	(9.98, 1.99)	(9.97, 1.99)	(1.36, 26.81)	(1.07, 28.11)
LogN(*μ*, 0.2)	(9.97, 1.99)	(9.98, 2.00)	(1.34, 26.79)	(1.06, 28.04)
LogN(*μ*, 0.4)	(9.99, 2.00)	(9.97, 1.99)	(1.31, 26.73)	(1.03, 28.05)
LogN(*μ*, ln(2))	(9.97, 1.99)	(9.97, 2.00)	(1.23, 26.66)	(0.99, 28.00)
LogN(*μ*, 0.8)	(9.98, 1.99)	(9.98, 1.99)	(1.22, 26.61)	(0.97, 27.92)
LogN(*μ*, 1.0)	(10.00, 1.99)	(9.97, 1.98)	(1.17, 26.57)	(0.94, 27.88)
LogN(*μ*, 1.2)	(10.00, 1.99)	(9.99, 2.00)	(1.14, 26.54)	(0.92, 27.87)

We conclude from the simulation experiments that there is no significant difference in results between exponential and lognormal LOS for Example I. This can be explained by the structure of the threshold and optimal policy. Both admit arriving patients if beds are available, except for a type 1 patient if there is only 1 bed available, and is therefore similar to an Erlang loss model. Example II shows some difference between the exponential LOS and lognormal LOS. However, the difference is very small and only becomes apparent to some extent for very small or relatively large values of *σ* ^2^.

### Small scale systems: bed allocation

At the level of a single unit, the patient population is often diverse. This diversity may be related to medical diagnosis, clinical pathway, urgency, or medical discipline for combined units (such as at an IC that is used by different disciplines). Since diseconomies of scale are large for small unit sizes, organizing dedicated beds for small patient groups should be avoided. Moreover, the medical staff in general can treat all patient types visiting the unit so disadvantages related to multi-skill workers are of minor concern. In terms of our bed allocation policies, a unit usually acts in practice as ‘simple merging’.

From the examples in Section 4.1, it follows that such a policy may not always deal well with different patient types in terms of prioritization and ALOS. To determine effective allocation policies, we now study the performance of bed allocations for a set of different problem instances. Since the optimal policy is hard to implement in practice, we compare it with the performance of simple merging, earmarking and the threshold policy. To this end, we generate 50 problem instances at random with the following specifications

- 2 types of patient classes;
- the number of beds *N*_*i*_ is uniformly distributed on [6,36];
- the average length of stay *β*_*i*_ is uniformly distributed on [1,14];
- the importance of a patient class *α*_*i*_ is uniformly distributed on [1,10]; thus, *α*_*i*_/*α*_*j*_ is the relative importance of class *i* compared to class *j*;
- the arrival rate of patients *λ*_*i*_ is such that the relative offered load (i.e., *λ*_*i*_ *β*_*i*_/*N*_*i*_) is uniformly distributed on [0.5,1.3].

**Fig. 3:**
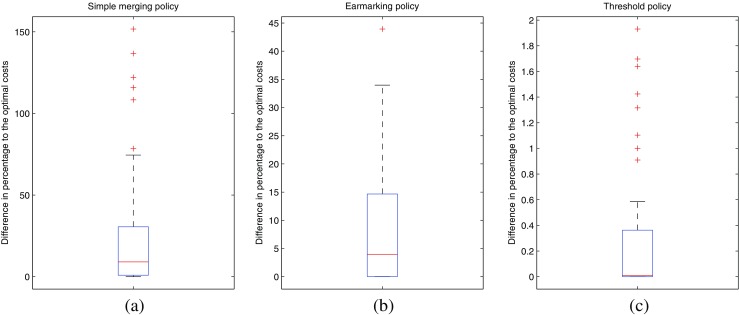
Relative difference in average costs for simple merging (**a**), earmarking policy (**b**) and threshold policy (**c**) compared to the average costs for the optimal policy

The performance is measured by comparing the costs of each policy to the optimal policy. Denote by *c* ^∗^ the optimal costs, and let *c* ^(*s*)^, *c* ^(*e*)^ and *c* ^(*t*)^ denote the costs associated with simple merging, earmarking, and the threshold policy, respectively. The performance is then calculated as (*c* ^(⋅)^−*c* ^∗^)/*c* ^∗^. Figure 3 shows boxplots of the 50 problem instances for the three policies. The boxes in the plots are bounded by the 25th and 75th percentiles, while the central mark is the median. The whiskers are the lower and upper adjacent values, respectively, that are within 1.5 times the interquartile range.

The results show that the threshold policy performs almost optimally. The maximum relative difference for the threshold policy is below 3.5 % compared to the optimal policy, and the average relative difference for all 50 problem instances is approximately 0.3 %. Simple merging is the worst among all studied policies with an average relative difference that equals 27 %. A huge benefit is obtained when we switch from simple merging to earmarking, with an average of the relative difference that is approximately 9 %. We note that the difference between simple merging and earmarking becomes larger when the load is larger. For instance, in case we take *λ*_*i*_ such that the relative offered load is uniformly distributed on [0.8, 1.3] the average relative differences are 0.4 %, 9 %, and 49 % for threshold policies, earmarking, and simple merging, respectively.

It is hard to say something about the situations in which a certain kind of policy performs well. From our numerical results, we have seen that the simple merging policy deviates more from the optimal policy as the difference between *ρ*_1_ and *ρ*_2_ increases. The same holds for the threshold policy. For the earmarking policy it turns out that the higher the difference between *α*_1_ and *α*_2_, the bigger the difference compared with the optimal policy.

#### **Conclusion for practice**

Threshold policies turn out to be effective for distinguishing between patient types. Moreover, the rules for admitting patients is relatively simple as it is based only on the number of available beds present at the unit. We therefore advocate to use policies of the threshold type. Our experience in practice is that doctors find it hard to reject patients when beds are still available. An exception might be the distinction between urgency classes, which is supported by medical staff.

### Large scale systems: bed distribution

The distribution of beds among different medical disciplines usually involves tens or hundreds of beds. The scenario of simple merging will then be infeasible in practice, as this would require all medical staff to be trained to treat all patient types. The threshold and optimal policy suffer from the same multi-skill problem. So, on a large scale separate beds for each patient class or earmarking allocations are the only feasible alternatives.

**Fig. 4:**
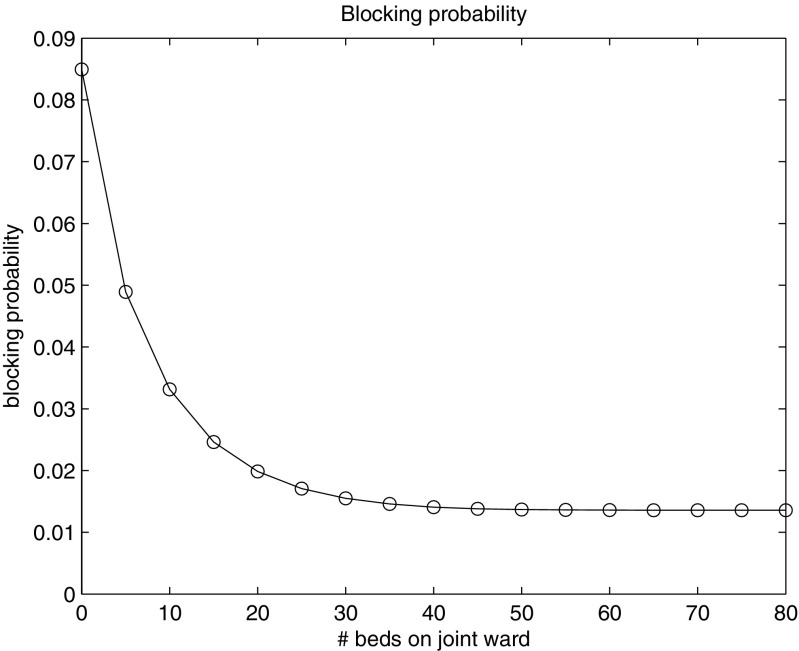
Blocking probability as a function of the number of flexible beds

For the earmarking policy, the shared or flexible beds may provide sufficient flexibility to utilize scale effects to a large extent. The lower part of Table 2 already suggested that some flexibility is sufficient for an efficient bed usage. As an illustrative example, consider the case of five symmetrical wards, each having a load of 20. Note that for the performance of any earmarking policy, only the load is required and not the specific arrival rate and ALOS. The total number of beds available for the five wards is 115. In Fig. 4 the blocking probability is displayed against the number of flexible beds (on the horizontal axis). If all beds are dedicated, then each ward gets 23 beds and the blocking probability is 8.49 %. When each ward allows only one bed to be flexible, resulting in 5 flexible beds overall, the blocking probability decreases to 4.89 %. Full flexibility, i.e. letting all 115 beds be flexible, results in 1.36 % blocking probability. As can be seen from Fig. 4, blocking probabilities below 2 % are already attained with 20 flexible beds. This illustration shows that a little flexibility is often sufficient to benefit from economies of scale. In particular, the blocking probability decreases as the number of flexible beds increases, but this happens in a convex way.

The illustration above indicates how many beds should be flexible. Another prime practical question is how to distribute beds across all units. This often is the most relevant issue as the total number of beds in the hospital is fixed, or changes in bed allocation should be such that the total number of beds remain fixed. Such questions can be explored by trying all combinations of bed allocations, but this number increases exponentially fast with the number of units in the hospital. Below, we identify guidelines for how many beds should be allocated to each unit. This allocation generally is a good starting point, but it may be tuned as there often are issues that are specific to local conditions. Examples of such conditions are construction of the building, nurse-to-patient ratios making it effective to be the number of beds being a multiple of some integer, historically obtained rights, policy considerations, etc.

The principle we propose for the distribution of beds is based on square-root staffing. Recalling that *ρ*_*i*_ is the offered load for unit *i*, the capacity should roughly be

3 $$ s_{i} = \rho_{i} + \beta_{i} \sqrt{\rho_{i}}, $$

for some $\beta _{i} \in \mathbb {R}$. For actual staffing the *s*_*i*_ need to be rounded. The first term *ρ*_*i*_ reflects that each unit should be able to handle the average offered load. The second term $ \beta _{i} \sqrt {\rho _{i}}$ represents the safety capacity, where *β*_*i*_ is the service level for unit *i*. Square-root staffing principles originate from heavy-traffic scenarios, but have turned out to be robust for smaller system sizes as well. We refer to, e.g., [3, 10, 11, 14, 32] for a more elaborate discussion. We now distinguish the cases with and without flexible beds; the bed allocation relies on square-root staffing for both cases.

#### **Bed allocation without flexible beds**

In the current situation, hospitals generally organize the clinic using separate wards. Admission of patients at other wards do occur, but this is commonly not organized on a structural basis. In Dutch hospitals it is common that admissions at non-preferred wards happen after rather exhaustive personal communications between medical supervisors of different medical units. As such, distribution of beds without organized flexibility is a prominent practical issue at the moment.

Using staffing rule (3), the blocking probability for unit *i* may be approximated by [14]

$$b_{i} = B(\rho_{i} + \beta_{i} \sqrt{\rho_{i}}, \rho_{i}) \approx \frac{\phi(\beta_{i})}{\Phi(\beta_{i}) \sqrt{\rho_{i}}}, $$

where *ϕ*(⋅) and Φ(⋅) are the density and cumulative distribution function of the standard normal distribution. The beds should now be allocated such that the sum of the capacities is *N* (see Eq. 4) and that the blocking probabilities satisfy the relative priorities (see Eq. 5). This yields the following system of *J* non-linear equations with as many unknowns *β*_*i*_, *i* = 1,…, *J*:

4 $$\begin{array}{@{}rcl@{}} \rho + \sum\limits_{j=1}^{J} \beta_{j} \sqrt{\rho_{j}} & = & N \end{array} $$

5 $$\begin{array}{@{}rcl@{}} \alpha_{1} \frac{\phi(\beta_{1})}{\Phi(\beta_{1}) \sqrt{\rho_{1}}} & = & \alpha_{i} \frac{\phi(\beta_{i})}{\Phi(\beta_{i}) \sqrt{\rho_{i}}}, \qquad i=2,\ldots,J. \end{array} $$

In extreme cases, this system of equations may be infeasible, for instance when blocking probabilities above 1 are required to satisfy relative patient values. In that case, it is recommended to carefully consider the specifications as such situations reflect unusual behavior in hospital operations. Otherwise, we opt to minimize the squared difference between the lhs and rhs of (5) constrained by (4).

As an illustration, we apply the concept above to a specific example. Consider 5 units representing, for instance, the different surgical disciplines. Let the load *ρ*_*i*_ and relative value *α*_*i*_ for each discipline be as given in Table 5. Hence, unit 5 is large, whereas units 2 and 4 have some preference over the other units. We note that blocking probabilities *b*_*i*_ are calculated using the continuous extension of the Erlang loss model, such that non-integral values of *s*_*i*_ can be taken into account.

**Table 5:** Bed allocation without flexibility

	load (*ρ*_*i*_)	rel. value (*α*_*i*_)	*β*_*i*_	*s*_*i*_	loss fraction (*b*_*i*_)
Unit 1	20	1	0.46	22.06	10.5 %
Unit 2	20	2	1.06	24.76	5.4 %
Unit 3	10	1	0.78	12.48	10.2 %
Unit 4	8	5	1.90	13.39	2.5 %
Unit 5	42	1	0.05	42.32	10.9 %

From Table 5 can be observed that the solution to the system of Eqs. 4 and 5 provides satisfying results and yields a good starting point to determine the final allocation. For the latter, we need at least rounding of *s*_*i*_.

#### **Bed allocation with flexible beds**

We assume that the number of flexible beds *M*_joint_ is given, and is not part of the allocation (otherwise, it could be beneficial to make almost all beds flexible as we did not consider costs for multi-skilled staff explicitly). This seems reasonable, as the decision on *M*_joint_ is typically influenced by many factors that are difficult to quantify. We note that the example above (Figure 4) provides a good intuition for appropriate choices of *M*_joint_.

Since there are now closed-form approximations for the blocking probability, we propose to use the following approximation scheme. Suppose that the flexible capacity is infinite. The number of type *i* patients in the system then has a Poisson distribution with mean *ρ*_*i*_, which is approximately normally distributed for *ρ*_*i*_ not too small. The probability that an arriving patient needs a flexible bed is then $\mathbb {P}(X_{i} \ge s_{i}) \approx 1 - {\Phi }(\beta _{i})$, with *X*_*i*_ the number of type *i* patients at an arbitrary arrival instant. The fraction of time that type *i* needs flexible beds should respect the relative value *α*_*i*_ between the different patient types. This is not precisely the same as the ratio between blocking probabilities, but the relative difference is typically small (unless the blocking probabilities are large).

The reasoning above leads to another set of *J* non-linear equations with as many unknowns *β*_*i*_, *i* = 1,…, *J* (see above in case this system of equations is infeasible). Again, the beds should be allocated such that the sum of the capacities is *N* (see Eq. 6) and that the fraction of time flexible beds are needed satisfy the relative priorities (see Eq. 7):

6 $$\begin{array}{@{}rcl@{}} \rho + \sum\limits_{j=1}^{J} \beta_{j} \sqrt{\rho_{j}} + {M_{\text{joint}}} & = & N \end{array} $$

7 $$\begin{array}{@{}rcl@{}} \alpha_{1} (1\,-\,{\Phi}(\beta_{1})) & \,=\, & \alpha_{i} (1\,-\,{\Phi}(\beta_{i})),\;i\,=\,2,\ldots,J. \end{array} $$

Consider the example above from Table 5, but now assume that it has been decided that 15 beds are flexible. The allocation of beds and the corresponding blocking probabilities *b*_*i*_ can be found in Table 6. Note that the blocking probabilities have decreased significantly compared to the situation without flexible beds. Units 1, 3, and 5 now have slightly less beds than their offered load.

**Table 6:** Bed allocation with 15 flexible beds

	load (*ρ*_*i*_)	rel. value (*α*_*i*_)	*β*_*i*_	*s*_*i*_	*N*_*i*_	loss fraction (*b*_*i*_)
Unit 1	20	1	–0.37	18.33	18	3.46 %
Unit 2	20	2	0.46	22.06	22	1.84 %
Unit 3	10	1	–0.37	8.82	9	3.14 %
Unit 4	8	5	1.13	11.20	11	0.88 %
Unit 5	42	1	–0.37	39.58	40	3.58 %

#### **Conclusion for practice**

Having some flexibility in bed usage is generally sufficient to cope with peaks in demand. As such, earmarking allocations are effective. Moreover, appropriate bed allocations can easily be supported with quantitative models. Our experience is that having fully flexible beds that are shared by all disciplines in hospitals are scarce (except for ICs or acute admission units). The same concept can also be carried out on a slightly smaller scale: related medical disciplines can partly share their beds according to an earmarking allocation. When the scale is large enough, such a cooperation is expected to perform well.

## Conclusion and discussion

In this paper we considered different practical alternatives to full flexibility of clinical beds or simple merging. The benefit of full flexibility can be easily explained by the economies of scale. However, full flexibility can be difficult to manage and may suffer from limited options of specialization in addition to issues in training many multi-skilled medical teams.

Our first contribution is that we propose structural and practically achievable bed allocation policies that perform well. For small scale systems, e.g., different patient groups at a medical unit, the benefits of a larger scale outweighs the drawbacks. To accommodate priorities of patient types and differences in lengths of stay, a threshold type of control is effective. In our numerical experiments we have seen that the threshold policy is nearly optimal, and in special cases coincides with the optimal policy.

For large scale systems, e.g., different medical disciplines, full flexibility is usually not desirable. However, a little flexibility is generally sufficient to benefit from most of the scale advantages. This can be implemented using an earmarking policy. Only a few members of the medical team need to be multi-skilled for little flexibility and yet the advantages are significant. In addition, we have addressed a prominent practical question of ‘how to distribute a fixed number of beds over different units?’. Using a square-root staffing principle, this can be efficiently determined by solving a set of equations.

The second contribution is that we provide models to support strategic and tactical decision making about the number of hospital beds. The performance analysis for earmarking is being implemented in a decision support system, exploiting the product-form solution, to facilitate hospital management in well-founded decisions about bed management.

From a practical point of view, we envisage that implementation of fair and flexible allocations is involved due to historically obtained privileges. Moreover, some specific characteristics of patient flows may be further explored to improve the accuracy of the model. For instance, in some situations, a delay model could be more appropriate than a loss model. Nonetheless, with the current models we display some key organizational concepts that are valid in a broader setting.

From a scientific standpoint, it is of future interest to find optimal bed allocations when costs are involved for single-skilled and multi-skilled medical teams (although it is not straightforward to quantify this in practice). Asymptotic regimes may give further theoretical support for the different bed allocations. Finally, extending the assumptions of the model could strengthen the conclusion.

## Footnotes

1 PASTA is the acronym for Poisson Arrivals See Time Averages, stating that the distribution of the number of customer seen by an arriving customers equals the time average distribution, see [33].

## References

[1] Altman E, Jiminez T, Koole GM (2001). On optimal call admission control in a resource-sharing system. IEEE Trans Commun.

[2] Bonald T (2006) Insensitive Queueing models for communication networks (2006). In: Proceedings of the Valuetools

[3] Borst SC, Mandelbaum A, Reiman MI (2004). Dimensioning large call centers. Oper Res.

[4] de Bruin AM, Bekker R, van Zanten L, Koole GM (2010). Dimensioning clinical wards using the Erlang loss model. Ann Oper Res.

[5] Burke EK, de Causmaecker P, Berghe GV, van Landeghem H (2004). The state of the art of nurse rostering. J Sched.

[6] Chevalier P, Shumsky RA, Tabordon N (2004) Routing and Staffing in Large Call Centers with Specialized and Fully Flexible Servers. Working paper, Simon School, University of Rochester, Rochester

[7] Green LV, Nguyen V (2001). Strategies for cutting hospital beds: The impact on patient service. Health Service Research.

[8] Green LV (2002). How many hospital beds?. Inquiry.

[9] Green LV (2005) Capacity planning and management in hospitals. In: Brandeau ML, Sainfort F, Pierskalla WP (eds) Operations research and health care, pp 15–41

[10] Gurvich I, Armony M, Mandelbaum A (2008). Service-level differentiation in call centers with fully flexible servers. Manag Sci.

[11] Gurvich I, Huang J, Mandelbaum A (2013). Excursion-based universal approximations for the Erlang-A queue in steady-state. Math Oper Res.

[12] Hall R (2012) Bed assignment and bed management. In: Hall R (ed) Handbook of healthcare system scheduling, pp 177–200

[13] Huckman RS, Zinner DE (2008). Does focus improve operational performance? Lessons from the management of clinical trials. Strat Manag J.

[14] Janssen AJEM, van Leeuwaarden JSH, Zwart AP (2008). Gaussian expansions and bounds for the Poisson distribution applied to the Erlang B formula. Adv Appl Probab.

[15] Kelly FP (1991). Loss networks. Ann Appl Probab.

[16] Koçağa YL, Ward AR (2010). Admission control for a multi-server queue with abandonment. Queueing Systems.

[17] Lippman SA (1975). Applying a new device in the optimization of exponential queueing systems. Oper Res.

[18] Lynck WJ (1995). The creation of economic efficiencies in hospital mergers. J Health Econ.

[19] Mandelbaum A, Momcilovic P, Tseytlin Y (2012). On fair routing from emergency departments to hospital wards: QED queues with heterogeneous servers. Manag Sci.

[20] Mandelbaum A, Reiman MI (1998). On pooling in queueing networks. Manag Sci.

[21] McManus ML, Long MC, Copper A, Mandell J, Berwick DM, Pagano M, Litvak E (2003). Variability in surgical caseload and access to Intensive Care services. Anesthesiology.

[22] Örmeci EL, Burnetas A, van der Wal J (2001). Admission policies for a two class loss system. Stoch Model.

[23] Rothkopf MH, Rech P (1987). Perspectives on queues: combining queues is not always beneficial. Oper Res.

[24] Smith DR, Whitt W (1981). Resource sharing for efficiency in traffic systems. Bell System Tech J.

[25] van Dijk NM, van der Sluis E (2004). To pool or not to pool in call centers. Prod Oper Manag.

[26] van Essen JT, van Houdenhoven M, Hurink JL (2014) Clustering clinical departments for wards to achieve a prespecified blocking probability. OR Spectrum, appeared online

[27] Vanberkel PT, Boucherie RJ, Hans EW, Hurink JL, Litvak N (2012). Efficiency evaluation for pooling resources in health care. OR Spectrum.

[28] Wallace RB, Whitt W (2005). A staffing algorithm for call centers with skill-based routing. Manufacturing and Service Operations Management.

[29] Walley P, Silvester K, Steyn R (2006). Managing variation in demand: lessons from the UK National Health Service. J Healthc Manag.

[30] Wolstenholme E (1999). A patient flow perspective of UK health services: exploring the case for the new “intermediate care” initiatives. Syst Dyn Rev.

[31] Worthington DJ (1987). Queueing models for hospital waiting lists. J Oper Res Soc.

[32] Whitt W (1992). Understanding the efficiency of multi-server service systems. Manag Sci.

[33] Wolff RW (1982). Poisson arrivals see time averages. Oper Res.

[34] Young JP (1965). Stabilization of inpatient bed occupancy through control of admissions. Journal of the American Hospital Association.

